# White Matter Tract Damage in the Behavioral Variant of Frontotemporal and Corticobasal Dementia Syndromes

**DOI:** 10.1371/journal.pone.0102656

**Published:** 2014-07-23

**Authors:** Fernanda Tovar-Moll, Ricardo de Oliveira-Souza, Ivanei Edson Bramati, Roland Zahn, Alyson Cavanagh, Michael Tierney, Jorge Moll, Jordan Grafman

**Affiliations:** 1 D'Or Institute for Research and Education (IDOR), Rio de Janeiro, Brazil; 2 Institute of Biomedical Sciences and National Center of Structural Biology and Bioimaging (CENABIO), Federal University of Rio de Janeiro, Rio de Janeiro, Brazil; 3 Institute of Psychiatry at King's College, De Crespigny Park, London, United Kingdom; 4 National Institute of Neurological Disorders and Stroke, National Institute of Health (NIH), Bethesda, Maryland, United States of America; 5 Rehabilitation Institute of Chicago, Chicago, Illinois, United States of America; University of Manchester, United Kingdom

## Abstract

The phenotypes of the behavioral variant of frontotemporal dementia and the corticobasal syndrome present considerable clinical and anatomical overlap. The respective patterns of white matter damage in these syndromes have not been directly contrasted. Beyond cortical involvement, damage to white matter pathways may critically contribute to both common and specific symptoms in both conditions. Here we assessed patients with the behavioral variant of frontotemporal dementia and corticobasal syndrome with whole-brain diffusion tensor imaging to identify the white matter networks underlying these pathologies. Twenty patients with the behavioral variant of frontotemporal dementia, 19 with corticobasal syndrome, and 15 healthy controls were enrolled in the study. Differences in tract integrity between (i) patients and controls, and (ii) patients with the corticobasal syndrome and the behavioral variant of frontotemporal dementia were assessed with whole brain tract-based spatial statistics and analyses of regions of interest. Behavioral variant of frontotemporal dementia and the corticobasal syndrome shared a pattern of bilaterally decreased white matter integrity in the anterior commissure, genu and body of the corpus callosum, corona radiata and in the long intrahemispheric association pathways. Patients with the behavioral variant of frontotemporal dementia showed greater damage to the uncinate fasciculus, genu of corpus callosum and forceps minor. In contrast, corticobasal syndrome patients had greater damage to the midbody of the corpus callosum and perirolandic corona radiata. Whereas several compact white matter pathways were damaged in both the behavioral variant of frontotemporal dementia and corticobasal syndrome, the distribution and degree of white matter damage differed between them. These findings concur with the distinctive clinical manifestations of these conditions and may improve the *in vivo* neuroanatomical and diagnostic characterization of these disorders.

## Introduction

The nosology of the frontotemporal dementias has been debated since they were recognized as distinct from the dementia of Alzheimer's disease on clinical, pathologic and genetic criteria [1]. One influential classification has sorted several non-Alzheimer dementia syndromes within the “Pick-frontotemporal complex” [2], of which the behavioral variant of frontotemporal dementia (bvFTD) and corticobasal syndrome (CBS) are principal types [3]. Although several studies have pointed to a specific cortical and subcortical pattern of atrophy in these neurodegenerative syndromes [4], the involvement of the white matter (WM), especially of its compact fiber tracts, needs to be further investigated.

Although these syndromes usually show specific cortical and subcortical patterns of atrophy, they also share regions of injury [4]. In particular, no study has directly compared WM abnormalities between CBS and bvFTD. Assessing the extension and pattern of WM damage in CBS and bvFTD may contribute to a deeper understanding of the anatomical network and clinicoanatomical correlations in dementias pertaining to the Pick-frontotemporal complex.

To date, relatively few studies have investigated the patterns of WM damage in subtypes of frontotemporal dementia with whole-brain tract-based spatial statistics (TBSS), a technique that offers improved reliability to the analysis and interpretation of multi-subject DTI datasets [5]. One recent investigation examining the pattern of WM damage in patients with frontotemporal dementia found widespread injury in the major commissural, association and projection tracts in comparison with Alzheimer's disease and normal controls [6]. However, most studies in this regard have used conventional DTI-derived measures instead. For example, Chao *et al*. [7] found that the regional pattern of WM loss was closely related to the regional loss of GM, suggesting a role for wallerian degeneration in the neuropathology of FTD. Differences of WM involvement in the frontal and temporal variants of FTD [8], as well as in bvFTD and primary progressive aphasia [9], have also been described. The distribution of WM damage in the prefrontal and temporal lobes in these conditions is consistent with the behavioral and neuropsychological symptoms of bvFTD [10]. The WM involvement in CBS has been even less explored. Most neuroimaging studies have limited the exam of WM to motor related pathways. Boelmans *et al*. [11], for example, found that the pyramidal tracts and the posterior trunk of the CC were altered in such cases. More recently, Sajjadi and colleagues studied 5 cases of CBS and proposed that at least some cases reflect diffuse WM disease only indirectly related to cortical degeneration [12]. Besides the obvious therapeutic implications of an early diagnosis of any dementing illness [13], the possibility that a subset of patients with bvFTD may not progress to dementia [14], and may even improve [15], provides additional impetus for the development of neuroimaging discriminators among Pick-frontotemporal dementia subtypes.

Here, we applied whole brain TBSS to the study of WM abnormalities in bvFTD and CBS. Given the extensive clinical and pathological overlap between bvFTD and CBS, we hypothesized that a direct comparison of these pathologies with whole-brain TBSS could reveal both distinctive and shared patterns of WM tract abnormalities. On the basis of previous functional and behavioral evidence [16], we predicted that (1) bvFTD would show greater WM abnormalities in fiber systems related to the prefrontal and anterior temporal lobes, while (2) CBS would show greater WM damage in the motor related pathways, as the body of the CC and the perirolandic projection pathways, especially the corticospinal tract. We finally predicted that (3) regional WM damage would be related both to common and specific symptoms of each condition.

## Materials and Methods

### Patients, Controls, and Cognitive and Neuropsychiatric Assessment

All subjects were seen as part of an ongoing research program on FTD and CBS in the Cognitive Neuroscience Section of the National Institute of Neurological Disorders and Stroke (NINDS) of the NIH, Bethesda, MD. They were either self-referred or referred by outside neurologists. Patients arrived at the NIH with a caregiver and were diagnosed based on an initial clinical evaluation and examination by standard clinical criteria. They then spent 9 days participating in extensive neuropsychological and neurologic testing and imaging studies. Twenty patients with a clinical diagnosis of bvFTD without motor neuron disease and 19 patients with a clinical diagnosis of CBS participated in the study. General inclusion criteria were strong right-handedness and English as first language. The bvFTD was diagnosed based on clinical and neuropsychological exams, and on visual inspection of MRI, following the consensus criteria of the Lund-Manchester Group [17] and the Work Group on Frontotemporal Dementia and Pick's Disease [18]. Patients with bvFTD presented with (i) progressive behavioral abnormalities with an early loss of insight as noted by caregivers and (ii) symmetrical or asymmetrical atrophy in the anterior temporal lobes, prefrontal lobes, or both, on MRI. Since we do not have neuropathological data on most patients we limited ourselves to making a clinical diagnosis of corticobasal *syndrome* and prudently refrained from making a diagnosis of corticobasal *degeneration*. A clinical diagnosis of CBS was made when dementia of insidious onset was associated with apraxia and an asymmetric akinetic-rigid syndrome; alien limb phenomena, myoclonus, and cortical sensory deficits supported the diagnosis [19]. Because structural imaging in CBS reflects clinical presentation rather than underlying pathology, MRI had lesser importance for the diagnosis in these cases than the clinical findings: it could show generalized cortical or frontoparietal atrophy, or even be normal for a particular age (for further details on the patient cohort, see references [20] and [21]). Unfortunately, we did not collect CSF and did not have access to PIB scans. Patients with a cardiac pacemaker, metallic implants or artifacts, unstable medical, endocrine and neurological disorders, and a history of a disabling psychiatric diagnosis (including cognitive, psychotic, or substance use disorder) were excluded. Fifteen healthy controls underwent an MRI in the same scanner after providing written informed consent. The study was approved by the National Institute of Neurological Disorders and Stroke Institutional Review Board. There were no statistically significant differences regarding gender, handedness, and education among the three groups (Table 1). We required all subjects to have an assigned research durable power of attorney prior to admission to the protocol and the assigned individuals, usually spouses, gave written informed consent for the study. The patients gave assent for the study. All aspects of the study and the consent procedure were approved by the NINDS Institutional Review Board. We might add that patients with FTD often have difficulty in assessing the meaningfulness or value of an activity due to their brain damage and therefore both durable power of attorney and assents were requested and approved by the NINDS Institutional Review Board.

**Table 1:** Clinical Features of Controls, bvFTD and CBS.

	Controls	bvFTD	CBS
N (M/F)	15 (8/7)	20 (10/10)	19 (10/9)
Age (years)* §	59.7±8.7	59.4±8.3	69.2±6.9
Education (years)	16.6±2.7	15.7±3.3	14.4±2.9
Handedness	15R	15R/4L/1RL	16R/3L
Duration of illness (years)	—	5.2±3.0	4.4±2.5
DRS-*2* Total score (0–144)	—	102±7	111±7
DRS-*2* Memory subscale (0–25)	—	16.6±5.7	20.1±5.1
DRS-*2* Construction subscale (0–6)*	—	4.5±0.5	2.2±0.5
NPI Total (0–144)*	—	37.3±15.2	6.2±6.7
NPI Apathy-D-AMB* (0–36)**	—	18.7±7.2	2.9±4.7
NPI Apathy (0–12)**	—	7.8±3.6	1.5±2.2
NPI Disinhibition (0–12)**	—	6.2±4.2	0.8±2.1
NPI Aberrant Motor Behavior (0–12)**	—	4.6±3.9	0.6±2.0

*Apathy-D-AMB: apathy-disinhibition-aberrant motor behavior.

**CBS ≠ FTD (Mann-Whitney *U* Test, *p*<0.05, two-tailed).

§ CBS ≠ Controls (Mann-Whitney *U* Test, *p*<0.05, two-tailed).

To increase the accuracy of a categorical diagnosis of bvFTD and CBS, global cognitive status and psychopathology were assessed, respectively, with the Dementia Rating Scale (DRS-*2*) and the Neuropsychiatric Inventory (NPI). The DRS-*2* was designed to assess the level of cognitive functioning in neurologically impaired individuals as shown by their performance on measures of Attention, Initiation-Perseveration, Construction, Conceptualization, and Memory [22]. The DRS-*2* gauges cognitive function at lower ability levels being sensitive to mild degrees of domain-specific impairments and is suitable for tracking changes in cognitive status over time. DRS-2 scores of our patients indicate that dementia was in its early stages in both groups, corresponding to a Clinical Dementia Rating stage of 1 [23]. The NPI is a 12-item instrument that assesses a range of psychopathological, “extracognitive”, symptoms that often occur in dementia, including delusions, hallucinations, dysphoria, anxiety, agitation, euphoria, disinhibition, emotional lability, apathy, and aberrant motor behaviors [24]. The NPI rates the information provided by an informant taking into account both the frequency and the severity of each abnormal behavior or psychopathological manifestation. Higher scores on the DRS-*2* and on the NPI imply, respectively, better cognition and worse psychopathology.

### Statistical analysis of demographic and clinical variables (Table 1)

Results are expressed as means and standard deviations (x±sd). The significance of the association between categorical variables was assessed with the *χ* ^2^ test. The significance of group differences for demographic and clinical variables was assessed with the Kruskal-Wallis test (*H*). The pairwise significance of differences was assessed *post hoc* with the Mann-Whitney (*U*) test. Spearman's correlation coefficients (*rho*, ρ) were computed in the patient group to investigate the strength of associations between DTI-derived metrics (FA and MD) and clinical scores as measured by the NPI (total and subscores of apathy, aberrant motor behavior and disinhibition) and Mattis DRB-2 (total scores and memory subscore). We set the level of significance (α) at 0.05, two-tailed, for all statistical tests. Effect sizes (*η*) of 0.10, 0.25, and 0.40, respectively, were accepted for small, medium, and large effect sizes; a statistical power (β)≥0.80 was accepted as satisfactory [25].

### Neuroimaging Data Acquisition, Processing, and Analysis

*Data acquisition*. Imaging data were obtained in a 3.0 Tesla Philips-Achieva scanner. In addition to standard spin-echo T1-weighted and turbo spin-echo T2-weighted anatomical sequences, diffusion-weighted images (DWI) were acquired in axial plane with a single-shot, spin-echo echoplanar sequences in the axial plane (TR/TE = 6000/85 ms., FOV = 240 mm, matrix  = 96×77 (reconstructed 128×128), slice thickness  = 2.5 mm without gap). Diffusion sensitization gradients were applied in 32 non-collinear directions, with a *b* factor of 1000 sec/mm^2^.

#### Diffusion tensor image post-processing

Prior to analysis, patients and control datasets were anonymized and randomized across subjects and groups. For each subject, and before estimating the specific diffusion maps, all diffusion images were visually inspected for artifacts. Non-diffusion and diffusion images were co-registered to correct for movement artifacts and eddy current distortion effects on EPI readout. The diffusion tensor for each voxel was calculated based on the eigenvectors (v_1_, v_2_, v_3_) and eigenvalues (λ_1_, λ_2_, λ_3_) using multivariate fitting and diagonalization. After the FA and MD maps were calculated from the eigenvalues, color-coded maps were generated from the FA values and three vector elements of v_1_ to visualize the WM tract orientation (FSL 4.0 FMRIB software) [26], [27]. FA and MD images were brain-extracted (Brain Extraction Tool, FSL) and registered to a common space (MNI152) using constrained nonlinear registration (Image Registration Toolkit) [27] (BET, DTIFit toolbox, part of FSL 4.0 FMRIB software). The derived FA and MD [26], [27], [28] data were further analyzed using *a priori* regions of interest (ROI) analysis and voxelwise whole-brain TBSS [5], [28].

#### ROI analysis

ROIs were placed by an experienced investigator using a DTI-MRI atlas of human WM for determining fiber tract orientation using ROIEditor/DtiStudio software (DtiStudio software) [26]. All ROIs were visually checked to confirm their location, to ensure that partial volume effects were minimized and that each ROI contained homogeneous fiber populations through examination of slices one dorsal and one caudal from the target. For each subject, fixed-size square ROIs (25 pixels) were outlined on the color-coded FA maps in the axial plane. The ROIs were placed along the corpus callosum (CC), genu (CC_genu_) and splenium (CC_spl_), bilateral posterior limb of internal capsule (IC), bilateral corona radiata (Cor_rad_), bilateral frontal (UF_front_) and temporal (UF_temp_) uncinate fasciculus, and anterior and mid-cingulate bundle (CB_ant_ and CB_mid_). The ROIs were automatically loaded onto the FA and MD maps and visually checked to confirm their location and ensure that partial volume effects were minimized. Means and standard deviations of FA and MD measures within each ROI were automatically recorded. For ROIs placed in the IC, Cor_rad_, UF_front_, UF_temp_, CB_ant_ and CB_mid_, mean right and left hemisphere FA and MD values were considered for further analysis. Possible interactions among groups and DTI-derived measures were assessed with a mixed ancova entering age as a covariate (*p*<0.05).

#### Voxelwise whole-brain analysis

To assess the global differences in the WM tracts between patients and controls and between CBS and bvFTD patients, whole-brain voxelwise statistical analyses of FA and MD were conducted using TBSS (FMRIB Software Library, FSL [29]). To preserve the intactness of WM structure and overall tracts as much as possible, a voxelwise specific-tuned nonlinear registration method was used to register FA and MD images into a standard space (IRTK software, Image Registration Toolkit [30]). FA images were aligned to the first subject FA image, which was taken as the reference target; this target image was then affine-registered into MNI152 standard and upsampled to 1×1×1 mm using both the non-segmented and segmented 1×1×1 mm MNIT1 brain volume; then, both the nonlinear and affine transformations from the target image were applied to the FA images of each subject. Resampled aligned FA images were averaged to create the mean FA from all subjects. The mean FA was then used to generate the “skeleton tract”, which represents the tracts shared by all subjects [5]. Finally, registered FA data from each subject were “projected” onto the mean FA skeleton mask to generate the final skeletonized FA data. Compared to controls, we expected WM abnormalities in both patient groups in the CC, as well as in six major intrahemispheric long association pathways: superior longitudinal fasciculus (SLF), inferior longitudinal fasciculus (ILF), superior fronto-occipital fasciculus (SFOF), inferior fronto-occipital fasciculus (IFOF), uncinate fasciculus (UF), cingulate bundle (Cing), and medial forebrain bundle (MFB), which was recently described in the human brain *in vivo* [31].

To test for significant local FA and MD differences among bvFTD, CBS, and controls voxelwise cross-subject statistical analysis was carried out using permutation-based non-parametric inference with 10,000 random permutations (FSL Randomise tool, [30] on each voxel of the resulting “mean skeletonised” data [13], [26] controlling for age. The results were considered significant at *p*<0.05, using cluster-based TFCE (Threshold-Free Cluster Enhancement [32] fully corrected for multiple comparisons (Familywise Error Rate, FWE). Voxels that differed from controls were compared between bvFTD and CBS. The thresholded-skeletonized resulting image was thickened for better visualization.

## Results

### Demographic and Clinical Results

The lack of significant differences in length of illness was a likely result of the later onset of dementia in the CBS group. Overall cognitive impairment did not statistically differ between the patient groups, but, as expected, psychopathology was more severe in bvFTD than in CBS due to higher degrees of apathy, disinhibition and aberrant motor behaviors in bvFTD. Although patients with CBS were significantly older than both controls and bvFTD patients, all differences remained statistically significant after covarying age in the relevant comparisons (Table 1).

### DTI ROI analysis

Comparisons between patients and controls showed statistically significant interactions between group and DTI-derived metrics in several regions. When compared to controls, patients showed lower FA, higher MD, or both in all investigated tracts, with exception of the IC and CC_spl_. Statistical power was higher than 0.85 and effect sizes were large for all significant effects. These values were remarkable for the DTI-derived measures that distinguished one group of patients from another. Thus, CBS patients presented with greater damage in the Cor_rad_, while bvFTD patients exhibited greater damage in the UF_front_, UF_temp_ and CB_ant_ (Table 2).

**Table 2:** Mean MD and FA values, statistical significance of group comparisons (α = 0.05, two-tailed), effect sizes (*η* ^2^)^a^ and statistical power (1−β)^b^.

ROI	DTI	CONTROLS	bvFTD	CBS	*η* ^2^	Power
**Differences among the three groups (NC ≠ bvFTD ≠ CBS)**						
Cor_rad_	FA	0.78600±0.03978	0.74850±0.044988	0.71473±0.04599	0.53	>0.97
UF_post_	FA	0.64066±0.02789	0.56750±0.053299	0.58736±0.02423	0.63	>0.99
**bvFTD different from NC and CBS (NC ≠ bvFTD; bvFTD ≠ CBS; NC ∼ CBS)**						
Cing_ant_	MD	0.00042±0.00005	0.00055±0.000098	0.00048±0.00005	0.54	>0.98
UF_ant_	MD	0.00039±0.00004	0.00046±0.000063	0.00042±0.00003	0.45	>0.87
UF_post_	MD	0.00044±0.00003	0.00056±0.000140	0.00049±0.00004	0.47	>0.90
**bvFTD different from NC (NC ≠ bvFTD; NC ∼ CBS; bvFTD ∼ CBS)**						
CC_genu_	FA	0.95866±0.01355	0.87650±0.084309	0.93000±0.03091	0.49	>0.91
**bvFTD and CBS different from NC (NC ≠ bvFTD; NC ≠ CBS; bvFTD ∼ CBS)**						
CC_genu_	MD	0.00042±0.00003	0.00054±0.000109	0.00048±0.00005	0.54	>0.97
Cor_rad_	MD	0.00034±0.00001	0.00036±0.000024	0.00037±0.00002	0.46	>0.88
Cing_ant_	FA	0.40733±0.06766	0.32315±0.036063	0.34842±0.03760	0.60	>0.99
Cing_post_	FA	0.69266±0.04605	0.63200±0.055402	0.63684±0.03902	0.51	>0.95
UF_ant_	FA	0.70533±0.04596	0.60750±0.078731	0.63157±0.06735	0.49	>0.93

ROI: regions of interest; DTI: diffusion tensor imaging; NC: normal controls; bvFTD: behavioral variant fronto-temporal dementia; CBS: corticobasal syndrome; Genu (CC_genu_) and splenium (CC_spl_) of the corpus callosum; bilateral posterior limb of internal capsule (IC); bilateral corona radiata (Cor_RAD_); bilateral frontal (UF_front_) and temporal (UF_temp_) sectors of the uncinate fasciculus; anterior and mid-cingulate bundle (CB_ant_ and CBmid); Fractional anisotropy (FA); mean diffusivity (MD); *η* ^2^: effect size; MD in mm^3^/s.

### DTI voxelwise whole-brain findings

**Figure 1:**
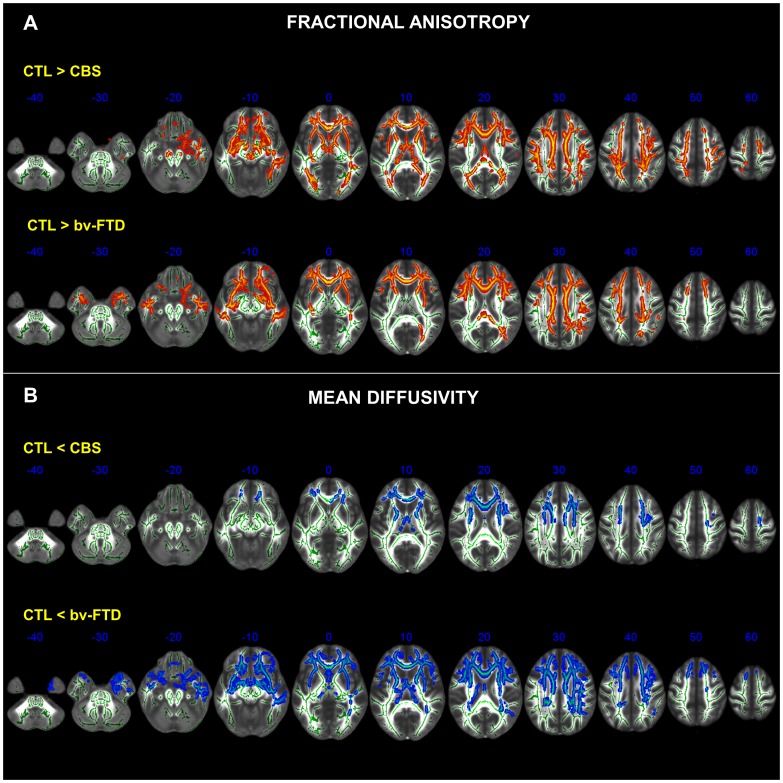
Clusters of voxels significantly different (*p*<0.05, FWE corrected for multiple comparisons across space using TFCE (Threshold-Free Cluster Enhancement) from healthy controls (CTL) are shown in red for FA (A) and in blue for MD (B) measures in the white matter of bvFTD and CBS patients. Each panel shows the significant voxel clusters superimposed on the mean FA map in the axial slices. MNI coordinates are shown. Images are displayed in radiological convention (right brain on left side).

**Figure 2:**
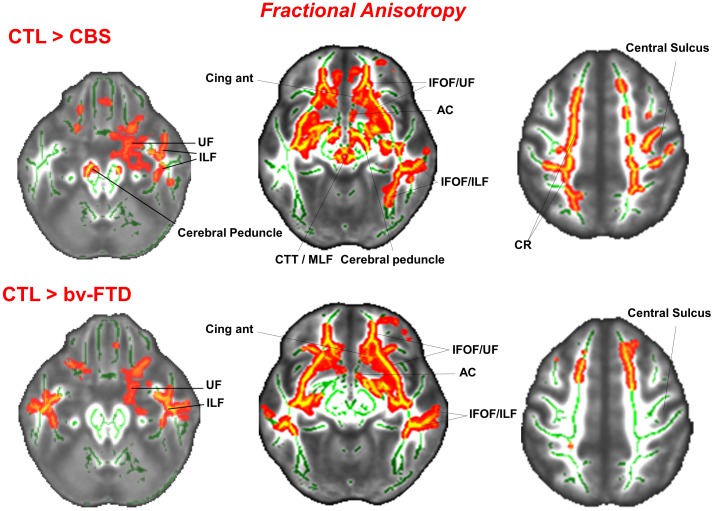
Clusters of voxels that differ significantly (*p*<0.05, FWE corrected for multiple comparisons across space using TFCE (Threshold-Free Cluster Enhancement) between normal controls (CTL) and CBS patients (upper row) and between normal controls and bvFTD patients (bottom row) for measures of FA in selected axial slices. MNI coordinates are shown. UF =  uncinate fasciculus; ILF =  inferior longitudinal fasciculus; AC =  anterior commissure; CingAnt  =  anterior cingulate; CTT/MLF =  central tegmental tract/medial longitudinal fasciculus; IFOF/ILF =  inferior fronto-occipital fasciculus/inferior longitudinal fasciculus; CR =  corona radiata.

In comparison to controls, both bvFTD and CBS showed varying degrees of roughly symmetrical abnormalities in the CC, anterior commissure, Cor_rad_, and in the long intrahemispheric association pathways, most notably the UF, Cing, IFOF, ILF, SFOF, SLF, and MFB. Degeneration of both projection (*i.e.*, corona radiata) and association pathways was clearly seen in the external and extreme capsules as well as in the supraventricular axial slices (Figure 1). In the bvFTD group, however, degeneration of the IC was confined to its anterior limb. The degeneration of the UF in the CBS group was more pronounced in the right hemisphere. Besides, while in CBS there were widespread abnormalities in the midbrain tegmentum and peduncle, as well as in the dorsal hippocampal commissure, such abnormalities were absent or much less severe in the bvFTD group (Figure 2).

**Figure 3:**
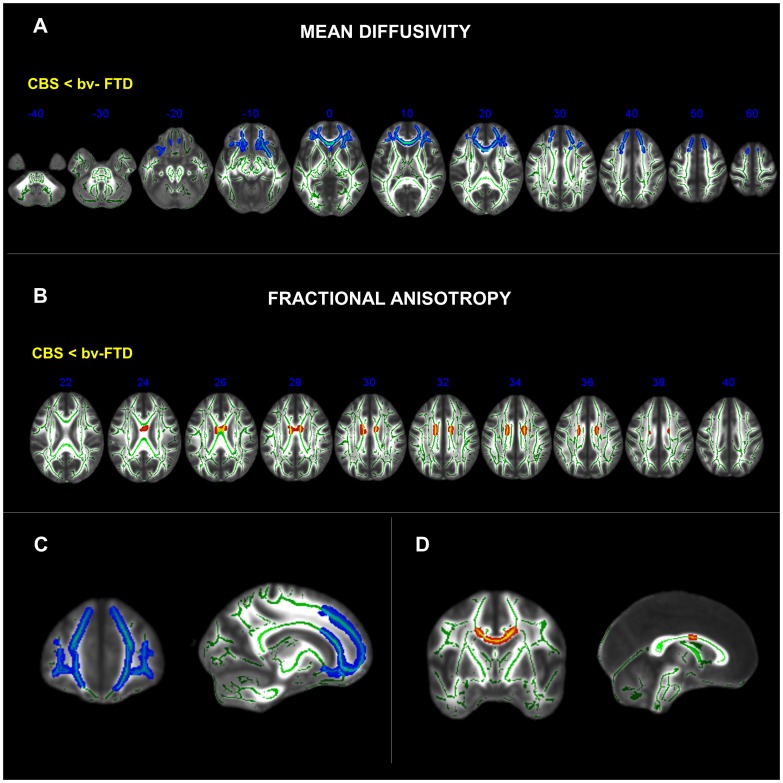
Clusters of voxels that differ significantly (*p*<0.05, corrected for multiple comparisons across space (FWE) using TFCE (Threshold-Free Cluster Enhancement) between CBS and bvFTD patients for measures of MD in blue and FA in red in the axial slices (A and B), and selected coronal and sagittal slices (C and D). MNI coordinates shown in the axial slices.

A direct comparison between patient groups revealed that, in comparison to the CBS group, the bvFTD patients showed a rostral region of symmetrical WM damage restricted medially to the forceps minor, continuing into the CC_genu_, UF, MFB, and rostral portion of the SLF. The midbody of the CC and the division of the Cor_rad_ related to the perirolandic cortex was more damaged in CBS than in bvFTD (Figure 3). When age was entered as a confounder in the statistical model, bvFTD showed a similar pattern of abnormality when compared to CBS. In this analysis, bvFTD was also associated with increased damage in the CC_genu_, UF and rostral portion of the SLF especially in the right hemisphere, when compared to the CBS patients.

**Figure 4:**
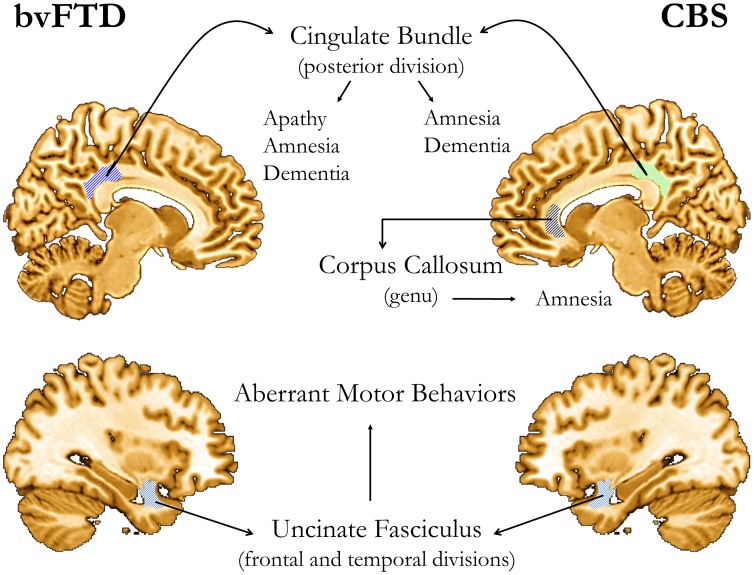
Summary of significant correlations between DTI (either FA or MD) and clinical measures. Cognitive (MDS-2) and psycho-pathological (NPI) abnormalities were related to focal WM damage in the posterior cingulate bundle, the uncinate fasciculus, and genu of the corpus callosum. (No laterality of findings implied by the depictions of the cerebral hemispheres).

*Correlations between DTI parameters and clinical ratings*. Significant correlations were found between the severity of symptoms and WM integrity in different brain regions. All the significant correlations were in the expected direction. *In bvFTD*, changes in posterior CB were correlated to severity in the NPI-apathy subscore (MD, ρ = 0.68, *p*<0.01), Mattis total (MD: *ρ* = −0.64, *p*<0.01; FA: ρ = 0.49, *p*<0.05) and Mattis-memory subscore (MD: ρ = −0.45, *p*<0.05; FA: ρ = 0.49, *p*<0.05), while severity in the NPI-aberrant motor behavior subscore was correlated with changes in the frontal (MD: ρ = 0.51, *p*<0.05; FA: ρ = −0.47, *p*<0.05) and temporal (FA: ρ = −0.48, *p*<0.05) segments of the UC fasciculus. *In CBS*, severity of symptoms measured by the Mattis-total score was correlated to changes in the posterior CB (MD: ρ = −0.76, *p*<0.01), while the Mattis-memory subscore was correlated to changes in the posterior CB (MD: ρ = −0.74, *p*<0.01) and genu of CC (MD, ρ = −0.53 *p*<0.05). In the same group, aberrant motor behavior was related to changes in the anterior (MD: ρ = −0.47, *p*<0.05) and posterior CB (FA: ρ = −0.48, *p*<0.05). (Figure 4).

## Discussion

This study confirmed the prediction (i) of the existence of a regional pattern of WM damage in bvFTD distinct from that of CBS, which was substantiated by robust statistical power and effect sizes, (ii) that damage to the WM tracts in each disease-condition was more widespread than previously thought, and (iii) that WM damage in discrete regions was related to both specific and common symptoms.

In agreement with the perirolandic and retrorolandic distribution of pathology, TBSS revealed in the CBS group a distinctive degeneration of the rostral brainstem (cerebral peduncle and tegmentum) and the anterior mediotemporal projections through the dorsal hippocampal commissure and fornix, which were absent or comparatively mild in bvFTD. The sectors of WM that interconnect homologous areas of the frontal poles through the anterior CC (genu and forceps minor), the frontal and temporal lobes (through the UF), and the anterior Cor_rad_, which sustains reciprocal projections between the PFC and the mediodorsal thalamic nucleus through the anterior thalamic radiation [33], were far more damaged in bvFTD than in CBS. One finding that sharply discriminated each patient group was a focal degeneration of the midbody of the CC in CBS. This region of the CC harbors the commissural fibers of the perirolandic cortices [34]. The CC degeneration that we found in CBS is consonant with the early perirolandic degeneration that is characteristic not only of CBD but also of those cases of Alzheimer's disease and progressive supranuclear paralysis that present clinically as CBS [35].

The pattern of WM damage in bvFTD and CBS concurs with the distribution of cortical pathology shown by *in vivo* neuroimaging [36] and *postmortem* studies [37]. The WM tracts that degenerate in bvFTD and CBS connect the frontal lobes with homologous areas of the contralateral hemisphere, as well as with the ipsilateral retrorolandic and temporal isocortices. Our results thus suggest the possibility that damage to WM pathways in bvFTD and CBS is more widespread than has so far been inferred from conventional DTI or even cortical volumetric measures [38], [39]. This higher yield may reflect the higher statistical power afforded by TBSS whole-brain WM analysis and by the increased signal-to-noise ratio provided by the higher field MRI scanners.

We also found that bvFTD and CBS share a common pattern of WM degeneration, thus providing further independent support for classifying these conditions within the same conceptual framework [3]. Besides the common regions that were affected in both pathologies, TBSS showed that CBS patients presented greater damage to the Cor_rad_, while bvFTD patients exhibited greater damage in the UF and anterior Cing, findings that were supported by large effect sizes.

The three focal regions of the WM with significant associations with clinical ratings were the posterior CB, the UF and the genu of CC. The posterior cingulate has been implicated both in the amnestic syndrome of Alzheimer's disease [40] and in apathy, with or without cognitive impairment, of diverse causes [41]. The CB contains fibers of different lengths and origins that may be as short as a U-fiber connecting two neighboring gyri or long enough to connect the temporal pole with the orbitofrontal cortex. It runs within the cingulate gyrus all around the CC up to the anteromedial temporal lobe, where it terminates [42]. Since the main conduction line of the CB is rostrocaudal, the damage observed in our cases occupies a strategic position to deprive the ventromedial temporal lobe of a massive input from different sources [43]. Deafferentation of ventromedial lobe structures such as the amygdala (emotional experience) and the hippocampus (episodic memory) may be instrumental in causing, respectively, apathy and amnesia. Damage to the UF, in turn, is consistent with the emergence aberrant motor behaviors, which is consistent with Starkstein and Robinson's model of disinhibition due to brain damage [44]. Finally, the validity of the association between rostral callosal damage and memory impairment or dementia is supported by observations on the classical forms of Marchiafava-Bignami disease [45].

This study has a number of limitations which should be addressed in the future. Although the older age of CBS patients may constrain some generalizations from our findings, an age-matched group of patients and controls in a study like this is unrealistic from an epidemiological point of view because of the histopathological heterogeneity of CBS and the earlier age of dementia onset of bvFTD [46]. Moreover, given the focus on TBSS we did not attempt to relate our findings to measures of regional cortical atrophy. Keeping these caveats in mind, the present findings suggest that the overall cognitive impairment observed in our patients may reflect the widespread pathology common to both bvFTD and CBS, whereas the impairment of specific subdomains of the DRS-*2* or the NPI results from the involvement of region specific cortico-subcortical circuits [21].
